# Transient Blockade of ERK Phosphorylation in the Critical Period Causes Autistic Phenotypes as an Adult in Mice

**DOI:** 10.1038/srep10252

**Published:** 2015-05-20

**Authors:** Shinya Yufune, Yasushi Satoh, Isao Takamatsu, Hiroyuki Ohta, Yasushi Kobayashi, Yumiko Takaenoki, Gilles Pagès, Jacques Pouysségur, Shogo Endo, Tomiei Kazama

**Affiliations:** 1 Department of Anesthesiology National Defense Medical College, 3-2 Namiki, Tokorozawa 359-8513, Japan,.; 2 Department of Physiology, National Defense Medical College, 3-2 Namiki, Tokorozawa 359-8513, Japan.; 3 Department of Anatomy and Neurobiology, National Defense Medical College, 3-2 Namiki, Tokorozawa 359-8513, Japan; 4 Institute for Research on Cancer and Aging (IRCAN), University of Nice Sophia Antipolis, Centre Antoine Lacassagne, 33 Avenue de Valombrose, Nice 06189, France; 5 Centre Scientifique de Monaco (CSM) Biochemical Department, 8 Quai Antoine Ier, MC 98000, Monaco; 6 Aging Regulation Research Team, Tokyo Metropolitan Geriatric Hospital and Institute of Gerontology, 35-2 Sakaecho, Itabashi-ku, Tokyo 173-0015, Japan

## Abstract

The critical period is a distinct time-window during the neonatal stage when animals display elevated sensitivity to certain environmental stimuli, and particular experiences can have profound and long-lasting effects on behaviors. Increasing evidence suggests that disruption of neuronal activity during the critical period contributes to autistic phenotype, although the pathogenic mechanism is largely unknown. Herein we show that extracellular signal-regulated protein kinases (ERKs) play important roles in proper formation of neural circuits during the critical period. Transient blockade of ERKs phosphorylation at postnatal day 6 (P6) by intraperitoneal injection of blood-brain barrier-penetrating MEK inhibitor, α-[amino[(4-aminophenyl)thio]methylene]-2-(trifluoromethyl)benzeneacetonitrile (SL327) caused significant increase of apoptosis in the forebrain. Furthermore, this induced long-term deleterious effects on brain functioning later in adulthood, resulting in social deficits, impaired memory and reduced long-term potentiation (LTP). Conversely, blockade of ERK phosphorylation at P14 no longer induced apoptosis, nor behavioral deficits, nor the reduced LTP. Thus, surprisingly, these effects of ERKs are strongly age-dependent, indicating that phosphorylation of ERKs during the critical period is absolutely required for proper development of brain functioning. This study provides novel insight into the mechanistic basis for neurodevelopment disorders: various neurodevelopment disorders might be generally linked to defects in ERKs signaling during the critical period.

As many have experienced, it is easier to learn a new language during the critical period than at other times. Accumulating evidence indicates that proper formation of neural circuits is influenced by experiences that take place in the critical period^1^. During the prenatal period, the basic layout of the neural circuit is established by genetic programs and innate activity. At birth, neurons have redundant synaptic connections not only to their proper targets but also to other cells. Then, during the critical period, neural circuits are refined under the influence of the real world (experience) by eliminating early-formed redundant synapses (called as synaptic pruning) and by selectively strengthening necessary connections^1^. The critical period closes after the consolidation of neural circuits. However, little is known about the mechanisms regulating formation of neural circuit during the critical period.

The critical period is a time of learning opportunity but also of vulnerability for interruption: interruption during the critical period could cause permanent and irreversible problems. It is well known that the closure of one eye (monocular deprivation) of a kitten during the critical period results in loss of visual acuity in the deprived eye despite no physical damage to the eye itself^2^. Neglected children exhibit severe developmental delay, mental retardation, and psychiatric symptoms^3^. In animal models, it was known that interruption of neural activity by low-dose of drugs could induce deleterious effects on neurons during the critical period. For instance, exposure to *N*-methyl-D-aspartate (NMDA) antagonist, ethanol, or general anesthetics, causes a significant increase in neuronal degeneration at P6–P7^4^,^5^,^6^. Furthermore, these interruption could cause deficits in brain functioning later in adulthood^6^, including social deficits similar to those seen in autistic spectrum disorder (ASD)^7^. Although little is known about the underlying mechanisms, it was reported that exposure to general anesthetics during the critical period induced transiently decrease of ERKs activity concomitant with neuro-apoptosis^8^. Furthermore, this neuro-apoptosis was attenuated by the counteractive effect of lithium on the ERKs suppression^8^.

ERKs are members of the mitogen-activated protein kinase (MAPK) family of Ser/Thr kinases. In neurons, ERKs play important roles in a diverse array of cellular processes (see review^9^). We previously reported that mice deficient for *Erk2* in the central nervous system (CNS) exhibited learning impairment and social deficits similar to those seen in ASD^10^. However, because *Erk2* undergoes conditional inactivation during the prenatal period in this mouse models, the role of ERKs during the critical period has been largely unknown.

In the current study, we hypothesized that ERKs play important roles in proper formation of neural circuits during the critical period, which is required for development of normal brain functioning. Therefore, we set out to examine the role of ERKs during the critical period.

## Results

### Inhibition of ERKs phosphorylation with MEK inhibitor caused apoptosis at P6 but not at P14

Studies about anesthetic-induced toxicity in brain development indicate that anesthetic-induced apoptosis is the greatest if exposure occurs at P6–P7^4^,^5^,^6^,^11^, with little or no increase in apoptosis at P14^11^ in rodents. Thus, there might be a critical period of vulnerability for the exposure to anesthetics with the peak at P6–P7 and the critical period would be closed before P14 in rodents. To determine the role of the ERK pathway in brain development during the critical period, we investigated whether the inhibition of ERK activation causes neurotoxicity in mice at P6 and P14.

**Figure 1:**
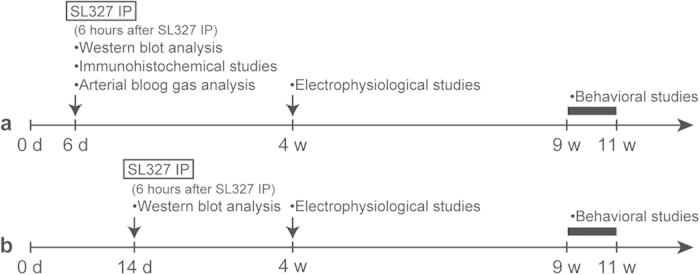
Experimental scheme detailing administration of SL327. **** (**a**) At postnatal day 6 (P6), mice were subjected to intraperitoneal (IP) injection with the MEK inhibitor, SL327 (50 mg/kg) or the same amount of vehicle (DMSO). At the end of 6-h period after administration, some groups of mice were sacrificed and assayed for apoptosis or arterial blood gas. Other groups were weaned and allowed to further mature. Then, they were either used for electrophysiological studies at 4 weeks of age or behavioral studies at 9-11 weeks of age. (**b**) In another experiment, mice were injected with SL327 (50 mg/kg) at P14. Then, some groups were assayed for apoptosis 6 h later similarly to mice administered SL327 at P6. Other group was weaned and allowed to further mature, and them, used for behavioral studies at 9-11 weeks of age.

**Figure 2:**
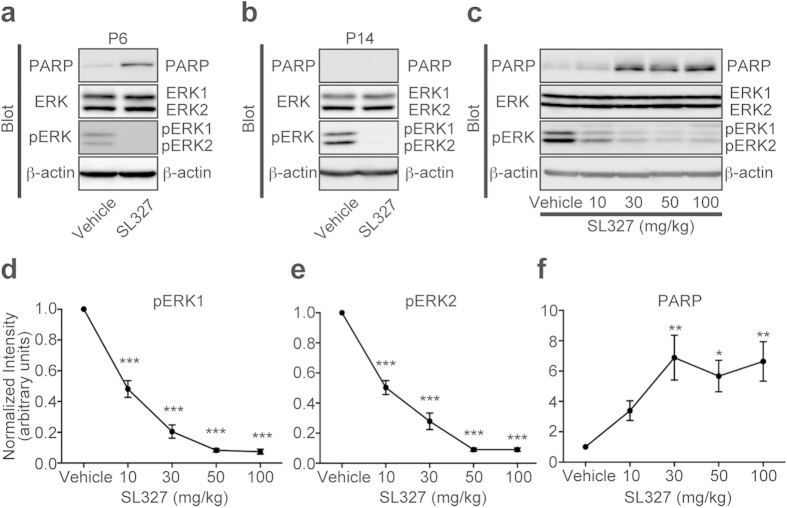
Transient SL327 administration causes apoptosis in the brain with strong age-dependency. **** (**a**) Representative images of western blot analysis using anti-cleaved PARP antibody showing that SL327 administration at P6 caused significant increase of apoptosis in the mouse compared with administration of vehicle (DMSO) (n = 10 for each). (**b**) Conversely, SL327 did not induce increased apoptosis at P14 (n = 6 for each). (**c**) Representative images of western blot analysis investigating the effect of each concentration of SL327 (0 (vehicle), 10, 30, 50, and 100 mg/kg). Expression levels of ERK1 and ERK2 were not significantly changed among concentrations tested. (**d**) The phosphorylation levels of ERK1 were decreased in a dose-dependent manner with saturation above 50 mg/kg of SL327. (**e**) The phosphorylation levels of ERK2 were decreased in a dose-dependent manner with saturation above 50 mg/kg of SL327. (**f**) SL327 concentrations above 30 mg/kg caused significant increase of apoptosis in the mice forebrain 6 h after administration. To evaluate expression and phosphorylation, band levels were divided by their corresponding loading internal control (β-actin). All the gels were run under the same experimental conditions. Cropped blots are shown in western blot data. Data are represented as mean ± SEM. *P < 0.05, **P < 0.01, ***P < 0.001 compared with vehicle controls (n = 7 mice for each concentration).

At P6, mice were intraperitoneally injected with the MEK inhibitor, SL327 (50 g/kg), and 6 h later brains were removed and assayed for apoptosis (Fig. 1a). It was previously reported that SL327 at this concentration can cross the blood-brain barrier and effectively reduce the basal level of ERK activation in the CNS^10^,^12^,^13^. We observed that single-dose administration of SL327 at this concentration effectively attenuated phosphorylation levels for ERK1 and ERK2 in the mouse forebrain at P6, compared with the vehicle group, by using an antibody for dually phosphorylated, and thus activated, ERKs (Fig. 2a). There was no concurrent decrease in total ERK levels for both isoforms. Using an antibody for cleaved PARP, we found that SL327 administration induced a significant increase in apoptosis in the brain compared with the vehicle group (Fig. 2a).

Next, we examined the effect of SL327 administration at P14 (Fig. 1b). We found no difference between the SL327-administered and vehicle group in levels of immunoreactivity for cleaved PARP, although the ERK phosphorylation levels were attenuated significantly by SL327 (Fig. 2b). These results indicate that the toxicity caused by the inhibition of ERK phosphorylation is strongly age-dependent.

We also determined the dose-dependent effect of SL327 administration on apoptosis (Fig. 2c). The phosphorylation of ERKs 6 h after SL327 administration reached a trough level above the concentration of 50 mg/kg SL327 (Fig. 2c–e). One-way ANOVA indicated a significant difference between SL327 concentrations (Fig. 2d, pERK1: *F* = 147.6, *P* < 0.0001; Fig. 2e, pERK2: *F* = 133.1, *P* < 0.0001). There was no concurrent decrease in total ERK levels for both isoforms with any of the SL327 concentrations tested (Fig. 2c). On the other hand, the levels of cleaved PARP reached the peak above the concentration of 30 mg/kg of SL327 (Fig. 2f). One-way ANOVA indicated a significant difference between SL327 concentrations (Fig. 2f; *F* = 5.80, *P* = 0.0011). Thus, the inhibition of ERK phosphorylation caused apoptosis in the developing brain in a dose-dependent manner.

**Figure 3:**
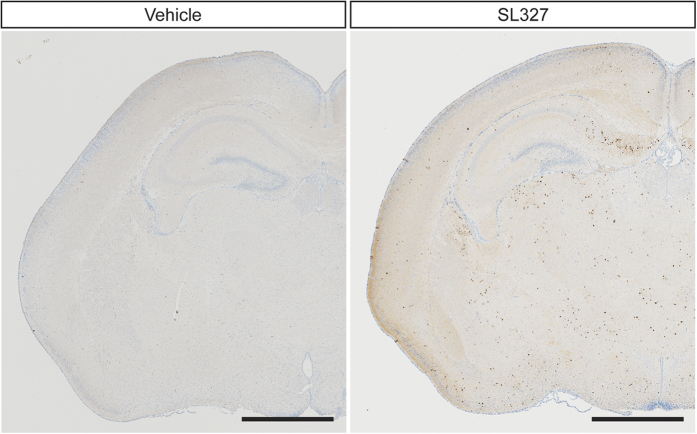
Number of black dots labeled by immunohistochemical staining for activated cleaved caspase-3 was increased in the brain from mice administered SL327 at P6. **** Representative light microscope views of coronal sections from the mouse brain 6 h after injection of vehicle or SL327. Black or brown dots represent immunostaining for activated caspase-3. High density of cleaved caspase-3 positive profile was present in mice administered SL327. Mouse brains were removed 6 h after injection. Scale bars: 1 mm.

**Figure 4:**
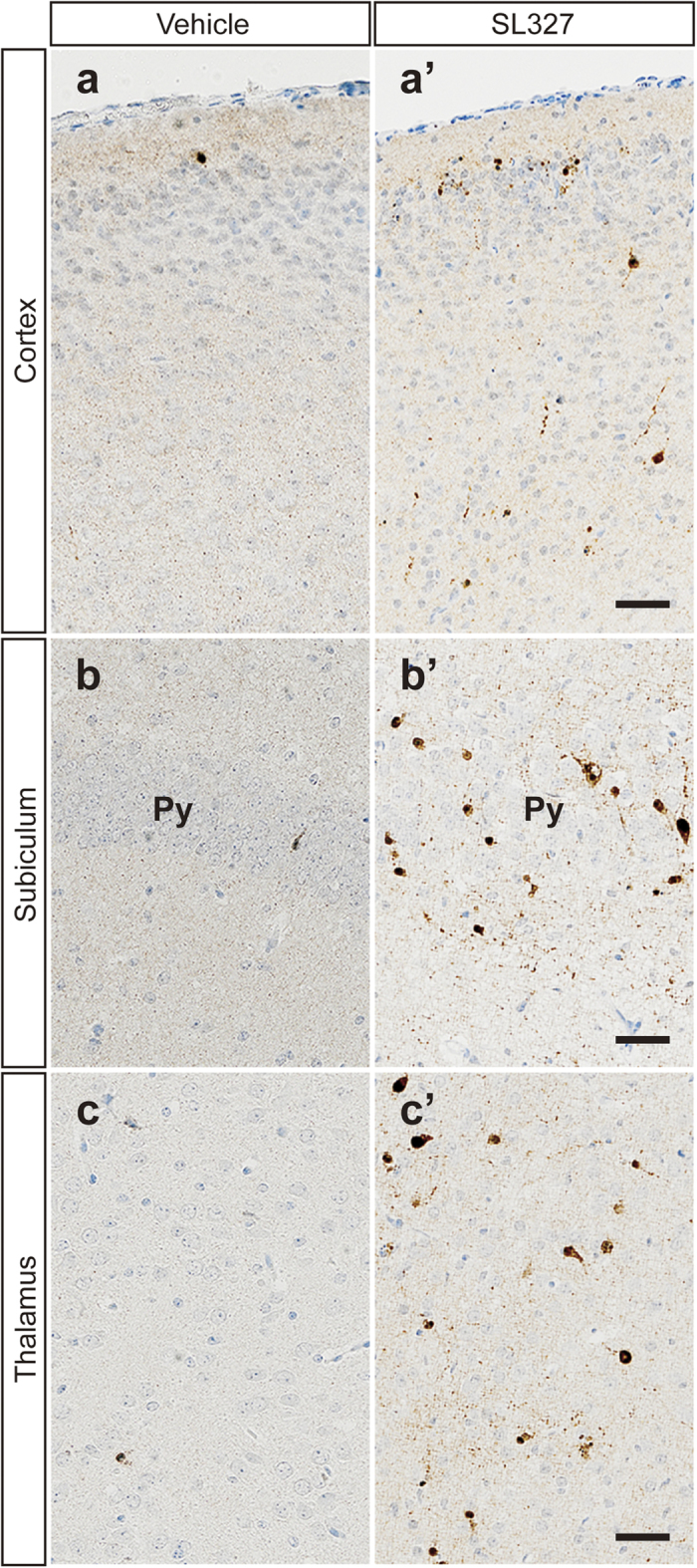
SL327 induced apoptosis in several regions of the brain at P6. **** Representative high-power images of the brains from mice treated with vehicle (**a**–**c**) or SL327 (**a’**–**c’**). (**a**,**a’**) cortex, (**b**, **b’**) subiculum, (**c**, **c’**) thalamus. Scale bars: 50 μm.

**Figure 5:**
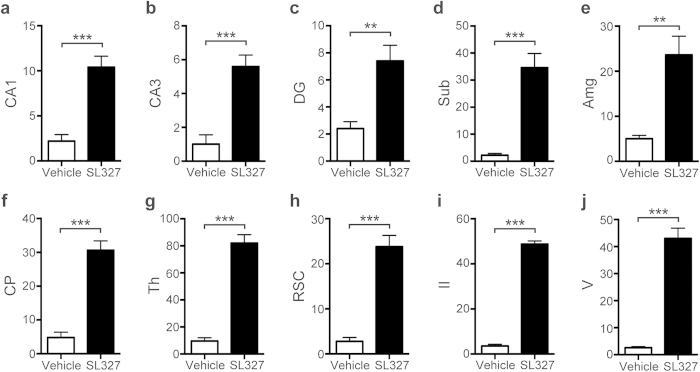
Quantification confirmed the significant increase of apoptosis in the brain from mice administered SL327 at P6 compared with vehicle controls. **** Numbers of cleaved caspase-3 positive cells. (**a**) CA1 = hippocampal CA1 region, (**b**) CA3 = hippocampal CA3 region, (**c**) DG = dentate gyrus, (**d**) Sub = subiculum, (**e**) Amg = amyg**d**ala, (**f**) CP = caudat**e**/putamen, (**g**) Th = thalamus, (**h**) RSC = retrosplenial cortex, (**i**) II = cortex layer II in parietal cortex, (**j**) V = cortex layer V in parietal cortex. Data are represented as mean ± SEM (n = 5 mice for each). **P < 0.01, ***P < 0.001.

**Figure 6:**
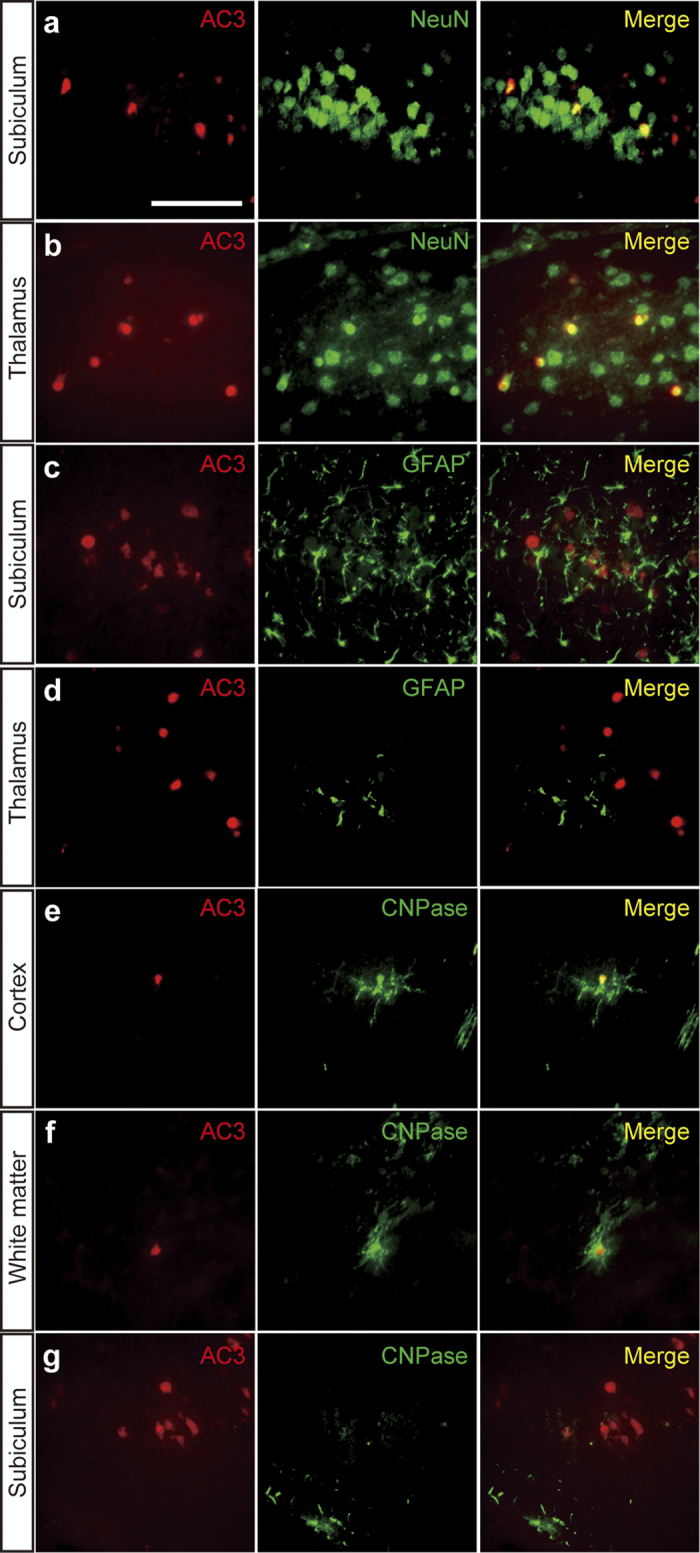
SL327 induced apoptosis mainly in neuron and oligodendrocyte. **** (**a**–**g**) Double immunostaining of activated caspase-3 with cellular markers for neuron (**a**, **b**), astrocyte (**c**, **d**), and oligodendrocyte (**e**–**g**). Activated caspase-3 signals were observed in neuron (**a**, **b**) but not in astrocyte 6 h (**c**, **d**) after SL327 injection at P6. Activated caspase-3 signals were also observed in oligodendrocytes in the cortex and white matter (**e**, **f**), but not in the subiculum (**g**). Scale bar: 50 μm.

Immunohistochemical analysis confirmed that the numbers of activated (cleaved) caspase-3^+^(AC3^+^) cells were significantly increased in mice treated with SL327 at P6 compared with those by natural apoptosis in vehicle controls (Fig. 3). Note that relatively well-preserved activated caspase-3^+^neurons, which may have been in an early stage of apoptosis, were seen occasionally (Fig. 4a’, b’ ). The quantification indicated significant differences in the numbers of AC3^+^cells between the SL327 and control group (Fig. 5). The types of AC3^+^cells were further examined by using the cell type-specific markers. Double staining for AC3 and the neuron-specific marker neuronal nuclear antigen (NeuN) revealed that apoptosis was induced in neurons (Fig. 6a,b). On the other hand, double staining for AC3 and the astrocyte-specific marker glial fibrillary acidic protein (GFAP) indicated that apoptosis was not induced in astrocytes after SL327 administration (Fig. 6c,d). Double staining for AC3 and the oligodendrocyte-specific marker 2’,3’cyclic nucleotide 3’ phosphodiesterase (CNPase) indicated that apoptosis was induced in oligodendrocyte as well as neurons in the cortex and white matter (Fig. 6e,f), although apoptosis was not observed in oligodendrocytes in the subiculum (Fig. 6g).

Analysis of arterial blood before and 6 h after SL327 administration revealed no significant differences in pH, partial pressure of arterial oxygen (PaO_2_), and partial pressure of arterial carbon dioxide (PaCO_2_) between the various groups (Table 1). Thus, SL327 administration did not have significant adverse effects on pH, PaO_2_, or PaCO_2_ compared with vehicle controls, suggesting that apoptosis caused by SL327 is not attributable to an indirect effect such as hypoxia.

### Administration of SL327 at P6, but not at P14, caused impairment of synaptic plasticity later in adulthood

**Figure 7:**
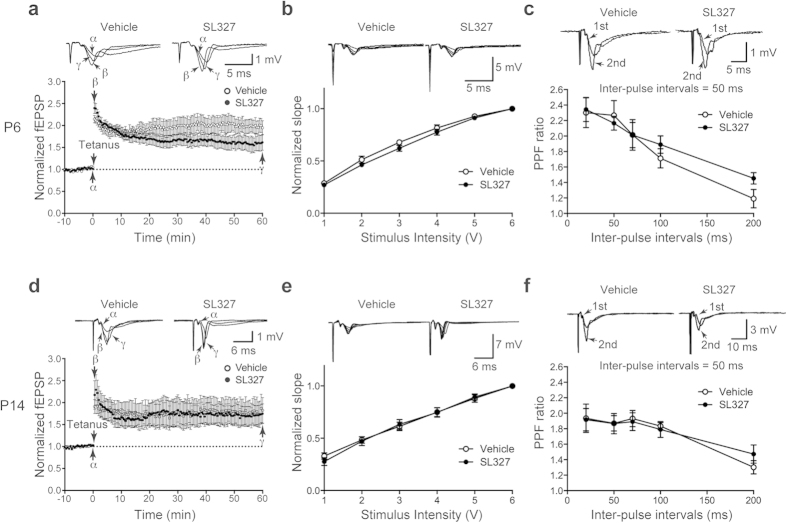
SL327 administration at P6, but not at P14, caused impaired long-term potentiation (LTP) at 4 weeks of age. **** (**a**–**c**) LTP was significantly suppressed in mice administered SL327 at P6 compared with controls (n = 6 mice for each). (**a**) Time course of change in field EPSP slope in hippoc**a**mpal slices from mice administered SL327 or DMSO (vehicle): (α) just before tetanus, (β) immediately after tetanus, and (γ) 60 min after tetanus. (**b**) Input-output function was not significantly changed in mice administered SL327 compared with controls. Traces of stimulus intensities are inserted in the upper panels. (**c**) PPF was not significantly changed in mice administered SL327 compared with controls. (**d**–**f**) LTP was not significantly suppressed in mice administered SL327 at P14 compared with controls (n = 6 mice for each). (**d**) Time course of change in field EPSP slope: α, β, and γ indicate time points as described in (**a**). (**e**) Input-output function was not significantly ch**a**nged in mice administered SL327 at P14 compared with controls. (**f**) PPF was not significantly changed in mice administered SL327 at P14 compared with controls. Data are represented as mean ± SEM.

To assess the long-term effects of neonatal administration of SL327 on neuronal function in modulating synaptic transmission, we investigated LTP at the Schaffer collateral-commissural pathway to CA1 pyramidal cell synapses, in hippocampal slices. Figure 7a indicates that neonatal administration of SL327 at P6 caused a profound suppression of LTP at 4 weeks of age compared with vehicle controls, despite the presence of robust short-term potentiation. A two-way repeated-measures ANOVA indicated a significant interaction between time and SL327 administration (*F* = 2.61, *P* < 0.0001), although no significant main effect of SL327 administration (*P* > 0.05). There was no significant change in the stimulus-response relationship and paired-pulse facilitation (PPF) between the SL327 group and the vehicle control group (Fig. 7b,c). These results indicate that SL327 administration during the critical period leads to impaired neuronal function in modulating synaptic transmission later in adulthood.

Conversely, we observed that LTP was not impaired at 4 weeks of age in mice administered SL327 at P14 compared with vehicle controls (Fig. 7d; two-way repeated-measures ANOVA, *P* > 0.05). There was no significant change in the stimulus-response relationship and PPF between the SL327 group and the vehicle control group (Fig. 7e,f).

**Figure 8:**
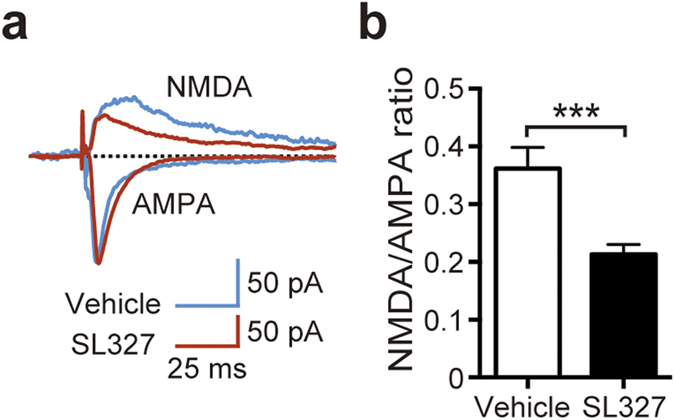
NMDA to AMPA ratio was altered in hippocampal neurons of mice administered SL327 at P6. (**a**) Representative traces from whole-cell voltage-clamp experiments showing NMDA receptor- and AMPA receptor-mediated currents recorded in a CA1 pyramidal neuron from mice administered SL327 (red) and vehicle (blue). Recording was performed at 4-5 weeks of age. (**b**) NMDA to AMPA ratio in mice administered SL327 and vehicle controls (control: n = 13, SL327: n = 15). Data are represented as mean ± SEM. ***P < 0.001.

Next, we compared NMDA-type glutamate receptor (NMDAR) current with AMPA-type glutamate receptor (AMPAR) current from the same cell (and stimulation). When recorded at 4-5 weeks of age, the ratio of NMDAR current to AMPAR current (NMDA/AMPA ratio) was reduced in mice administered SL327 at P6 compared with vehicle controls (Fig. 8; t-test, t = 3.85, *P* = 0.0007), suggesting deranged function of NMDA and/or AMPA receptor subunits at the postsynaptic sites in hippocampal neurons.

### SL327 administration at P6, but not at P14, caused abnormal behaviors later in adulthood

**Figure 9:**
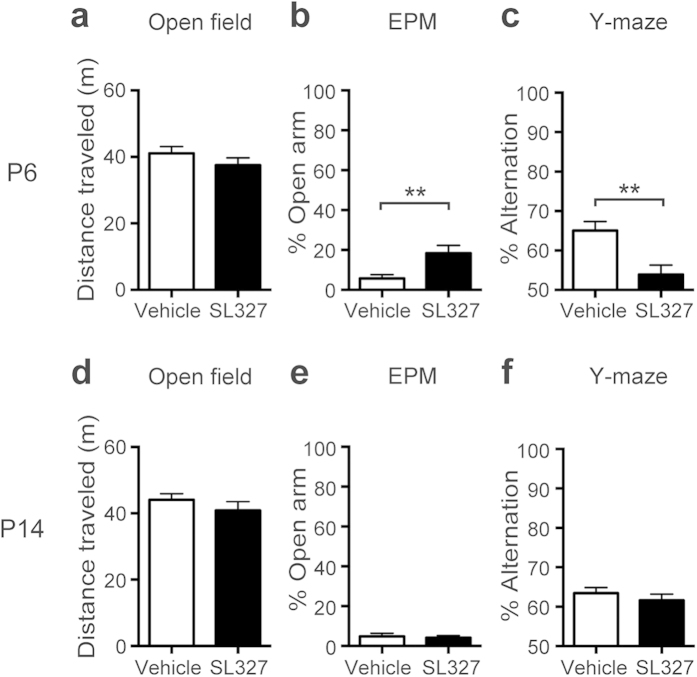
SL327 administration at P6, but not at P14, induced long-term deleterious effects, resulting in abnormal behaviors later in adulthood. **** (**a**–**c**) SL327 administration at P6 induced abnormal behaviors when assessed at 11 weeks of age. (**a**) No significant differences were observed in total distance traveled over 10 min in the open-field test (control: n = 14, SL327: n = 15). (**b**) Significant differences were observed in time spent in open arms in the elevated plus-maze (EPM) test (control: n = 13, SL327: n = 14). (**c**) Short-term spatial working memory was impaired in SL327-treated mice. Percentages of correct alternation responses on the Y-maze test are shown (control: n = 14, SL327: n = 15). (d–f) SL327 administration at P14 induced abnormal behaviors when assessed at 11 weeks of age. (**d**) No significant differences were observed in total distance traveled in the open-field test (control: n = 12, SL327: n = 14). (**e**) No significant differences were observed in time spent in open arms in the EPM test (control: n = 12, SL327: n = 14). (**f**) Short-term spatial working memory was not impaired in SL327-treated mice (control: n = 12, SL327: n = 14). Data are represented as mean ± SEM. **P < 0.01.

Next, we investigated whether SL327 administration at P6 induced behavioral changes later in adulthood (9-11 weeks of age). To examine responses to a novel environment, mice were assayed for the open-field test. No aberrant activity was observed in SL327-treated mice compared with vehicle controls as assessed by the total distance traveled (Fig. 9a; t-test, *P* > 0.05). To examine anxiety-related behavior, mice underwent the elevated plus-maze (EPM) test. Anxiety-related behavior was assessed based on the percentage of time spent in the open arms. Mice administered SL327 at P6 exhibited a significant increase in time spent in the open arm compared with vehicle controls, indicating reduced anxiety-related behavior (Fig. 9b; t-test, t = 2.85, *P* = 0.0087). This result is similar to those seen in mice that are deficient for *Erk2* in the CNS^10^. It should be noted that, in rodents, anxiety-related behavior in the EPM test reflects the natural balance between the exploratory and escaping drives, which would be conceptualized as risk-taking behavior. Thus, it is different from anxiety in humans, and increased time spent in the open arm might indicate the impaired recognition of the risk in these mice. To further examine whether SL327 administration at P6 induced impairment in memory function, mice were assessed for the Y-maze spontaneous alternation task. Mice administered SL327 at P6 performed this task significantly worse compared with vehicle controls, as assessed by the ratio of correct choice (Fig. 9c; t-test, t = 3.36, *P* = 0.0023). The mean value of vehicle controls was well above the expected result of random choices (random choice=50%; one-sample t-test, t = 6.63, *P* < 0.0001). Conversely, the mean value from mice administered SL327 at P6 was not significantly different from the expected result from random choices (random choice=50%; one-sample t-test, *P* > 0.05). Thus, spatial working memory was impaired by SL327 administration at P6.

Conversely, we observed that these behaviors were not impaired in mice administered SL327 at P14. In the open field test at 11 weeks of age, no aberrant activity was observed in SL327-treated mice compared with vehicle controls as assessed by the total distance traveled (Fig. 9d; t-test, *P* > 0.05). In the EPM test, no significant differences were observed in time spent in the open arms between mice administered SL327 at P14 and vehicle controls, indicating normal anxiety-related behavior (Fig. 9e; t-test, *P* > 0.05). In the Y-maze test, no significant differences were observed in the ratio of correct choice between mice administered SL327 at P14 and vehicle controls (Fig. 9f; t-test, *P* > 0.05). The mean values of vehicle controls and mice administered SL327 at P14 were well above the expected result of random choices (random choice=50%; one-sample t-test, control: t = 9.61, *P* < 0.0001, SL327: t = 7.40, *P* < 0.0001). Thus, spatial working memory was normal in mice administration SL327 at P14.

**SL327 administration at P6, but not at P14, caused social deficits later in adulthood**

**Figure 10:**
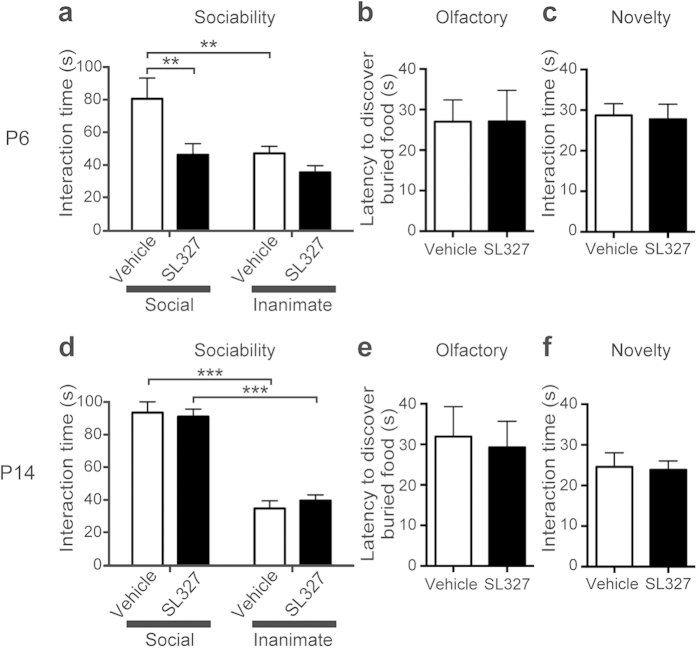
SL327 administration at P6, but not at P14, induced social deficits later in adulthood. **** (**a**–**c**) SL327 administration at P6 induced social deficits when assessed at 11 weeks of age (control: n = 13, SL327: n = 14). (**a**) Social behavior was assessed by the sociability test. When exposed to caged social and inanimate targets, controls exhibited a normal preference for the social target over an inanimate target, whereas mice with SL327 administration exhibited no significant preference. Furthermore, these mice spent significantly less time interacting with the social target compared with vehicle controls. (**b**) Mice treated with SL327 did not show significant differences from controls in latency to find a buried treat after overnight food deprivation. (**c**) Time spent interacting with a novel inanimate object was not significantly affected by SL327 treatment. (d–f) SL327 administration at P14 did not induce social deficits when assessed at 11 weeks of age (control: n = 12, SL327: n = 14). (**d**) Mice treated with SL327 exhibited normal behavior in the sociability test. (**e**) Latency to find a buried treat after overnight food deprivation. (**f**) Time spent interacting with a novel inanimate object. Data are represented as mean ± SEM. **P < 0.01, ***P < 0.001.

Next, we investigated whether SL327 administration would induce impairment of social behavior. In the sociability test, we examined the preference for interaction with animate (caged adult mouse) *versus* inanimate (caged dummy mouse) targets, assessed by interaction time. The vehicle control group spent significantly more time interacting with the social target than with the inanimate target (Fig. 10a). However, mice administered SL327 at P6 exhibited decreased interaction with the social target compared with controls. In SL327-administered mice, there was no significant difference between interacting with a social target and interacting with an inanimate target (Fig. 10a). A two-way ANOVA indicated a significant main effect of target (*F* = 8.37, *P* = 0.0056) and a significant main effect of SL327 administration (*F* = 9.05, *P* = 0.0041). We did not attribute the differences in social interaction to impaired olfactory sensation or loss of general interest in novelty, because we did not detect significant difference between the groups in tests for olfaction (Fig. 10b; t-test, *P* > 0.05) or for interaction with novel inanimate objects (Fig. 10c; t-test, *P* > 0.05). Therefore, it can be concluded that SL327 administration at P6 induced abnormal social behavior.

Conversely, we observed that social behavior was not impaired in mice administered SL327 at P14. In the sociability test, no aberrant activity was observed in SL327-treated mice compared with vehicle controls (Fig. 10d). A two-way ANOVA indicated a significant main effect of target (*F* = 127.6, *P* < 0.0001), but no significant main effect of SL327 administration (*P* > 0.05). We did not detect a significant difference between the groups in tests for olfaction (Fig. 10e; t-test, *P* > 0.05) or for interaction with novel inanimate objects (Fig. 10f; t-test, *P* > 0.05). Therefore, it can be concluded that SL327 administration at P14 did not induce abnormal social behavior.

**Figure 11:**
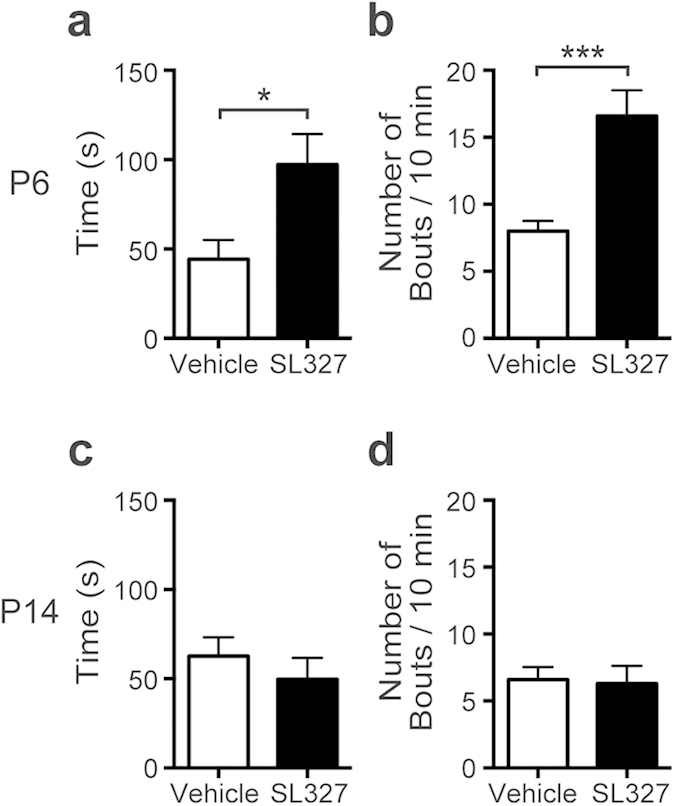
SL327 administration at P6, but not at P14, caused increased grooming behavior later in adulthood. **** (**a**, **b**) SL327 administration at P6 caused increased grooming behavior when assessed at 9 weeks of age (control: n = 10, SL327: n = 10). (**a**) Time spent self-grooming. (**b**) Number of grooming bouts. (**c**, **d**) SL327 administration at P14 did not cause increased grooming behavior when assessed at 9 weeks of age (control: n = 10, SL327: n = 10). (**c**) Time spent self-grooming. (**d**) Number of grooming bouts. Data are represented as mean ± SEM. **P < 0.01.

Because impairments in social behavior is one of hallmarks of ASD^14^,^15^, these results suggest that mice administered SL327 at P6, but not at P14, would have ASD-like symptoms later in adulthood. Thus, we next examined self-grooming, a stereotyped pattern of behavior because “restricted repetitive and stereotyped pattern of behavior” are other important behavioral domains in ASD^14^. Mice administered SL327 at P6 spent almost twice as much time grooming themselves as controls (Fig 11a; t-test, t = 2.60, *P* = 0.018) and showed increased frequency of grooming bouts (Fig 11b; t-test, t = 4.18, *P* = 0.0006). Conversely, mice administered SL327 at P14 did not exhibit significant alteration compared with controls (Fig 11c, d; t-test, *P* > 0.05). These results further suggest that mice administered SL327 at P6, but not at P14, have ASD-like symptoms later in adulthood.

### Blockade of both ERK isoforms was needed to induce apoptosis at P6

To examine the relative roles of each isoform of ERK, we further examined the effect of SL327 on the levels of cleaved PARP in *Erk1* knockout (KO) and *Erk2* conditional knockout (CKO) mice. *Erk2* CKO mice were generated by mating mice carrying *Erk2* floxed allele with those expressing the Cre recombinase under the control of the Nestin promoter, and resultant mice are deficient for *Erk2* specifically in the central nervous system^10^. We used *Erk2* CKO mice in the current study because mice with ubiquitous deletion of *Erk2* are embryonically lethal^10^,^16^.

**Figure 12:**
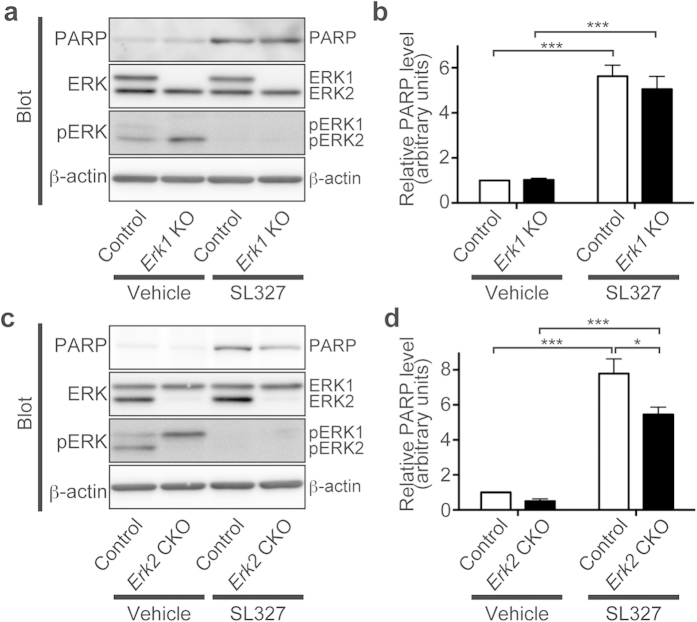
Blockade of both ERK isoforms was needed to induce apoptosis at P6. **** (**a**) Representative images of western blot analysis investigating the effect of SL327 administration at P6 in *Erk1* KO mice. (**b**) No significant difference was observed in the effect of SL327 administration on cleaved PARP levels in *Erk1* KO mice compared with those of controls (*Erk1* KO mice (SL327): n = 4, controls (SL327): n = 6). There was no significant difference on basal levels of cleaved PARP between *Erk1* KO mice and controls (*Erk1* KO mice (vehicle): n = 5, controls (vehicle): n = 5). (**c**) Representative images of western blot analysis investigating the effect of SL327 administration at P6 in *Erk2* CKO mice. (**d**) Cleaved PARP levels caused by SL327 administration at P6 were significantly lower in *Erk2* CKO mice compare**d** with those of controls (*Erk2*  CKO mice (SL327): n = 6, controls (SL327): n = 6). There was no significant difference in basal apoptosis levels between *Erk2*  CKO mice and controls (*Erk2*  CKO mice (vehicle): n = 5, controls (vehicle): n = 5). To evaluate the expression and phosphorylation, band levels were divided by their corresponding loading internal control (β-actin). All the gels were run under the same experimental conditions. Cropped blots are shown in western blot data. Data are represented as mean ± SEM. *P < 0.05; ***P < 0.001.

When vehicle was administered, there was no significant difference in levels of cleaved PARP between *Erk1* KO and control mice, indicating that the genetic abrogation of *Erk1 per se* does not cause increased apoptosis at P6 (Fig. 12a). In the presence of SL327, the level of apoptosis was increased in *Erk1*  KO mice to the extent that was comparable to those in control mice (Fig. 12a). A two-way ANOVA indicated no significant main effect of *Erk1* abrogation (*P* > 0.05), although there was a significant main effect of SL327 administration (Fig. 12b; *F* = 77.22, *P* < 0.0001). Post hoc testing revealed no significant difference in levels of cleaved PARP between *Erk1*  KO and control mice both in the presence of vehicle or SL327. Thus, we had hypothesized that abrogation of ERK2 would have a predominant effect in the induction of apoptosis at P6.

However, no significant difference was observed in the level of cleaved PARP between *Erk2* CKO and control mice when vehicle was administered (Fig. 12c), indicating that genetic abrogation of *Erk2 per se* did not cause increased apoptosis at P6, contrary to our expectation. Furthermore, in the presence of SL327, the level of apoptosis was increased in *Erk2* CKO mice, although the apoptosis level was significantly decreased when compared with control mice (Fig. 12d). A two-way ANOVA indicated a significant main effect of SL327 administration (Fig. 12d; *F* = 76.06, *P* < 0.0001), a significant main effect of *Erk2* abrogation (*F* = 7.76, *P* = 0.012), and a significant interaction between them (*F* = 4.48, *P* = 0.049). Post hoc testing indicated no significant difference in levels of cleaved PARP between *Erk2* CKO and control mice when vehicle was administered. On the other hand, post hoc testing revealed a significant difference in levels of cleaved PARP between *Erk2* CKO and control mice when SL327 was administered. These results indicated that blockade of both isoforms of ERK is needed to induce apoptosis at P6. The reduced level of cleaved PARP in *Erk2* CKO mice in the presence of SL327 compared with control mice was presumably due to developmental changes before P6.

## Discussion

In the current study, we found that transient blockade of ERKs activation caused robust apoptosis in the mouse brain at P6, but not at P14. Furthermore, SL327 administration at P6, but not at P14, caused long-term deleterious effects on neuronal functioning, resulting in cognitive impairments, social deficits, and altered repetitive behavior later in adulthood. This strong age-dependency suggests that the critical period closes between P6 and P14 in this mouse model. These results indicated that ERK phosphorylation should be required to ensure the proper development of neural functions during the critical period. We found that LTP was impaired in mice administered SL327 at P6. On the other hand, no difference of the PPF of fEPSPs was observed between mice administered SL327 and vehicle controls, indicating that the inhibition of ERK pathway leaves short-term presynaptic plasticity intact. Analysis of the input-output curve revealed no significant changes in the relationship between the stimulus intensity and the fEPSP slope in SL327-administered mice. Thus, basal synaptic transmissions appear to be unaltered in mice administered SL327 at P6. We also found that the NMDA/AMPA ratio was altered in mice administered SL327 at P6, indicating the deranged function of NMDA and/or AMPA receptor subunits at the postsynaptic sites might contribute to the reduced LTP, although the precise mechanism is still unknown. Further experiments are necessary to delineate the precise mechanism underlying alteration of the NMDA/AMPA ratio.

We found that increased apoptosis was observed mainly in neurons and oligodendrocytes, but not in astrocytes, when SL327 was administered at P6. Similarly, general anesthetics and alcohol reportedly caused apoptotic death mainly in neurons and oligodendrocytes, but not in astrocytes^17^,^18^. Thus, our results do not contradict the hypothesis that ERK pathway is, at least partly, involved in the mechanisms underlying apoptotic action of general anesthetics in the developing brain. We observed that oligodendrocytes in white matter and cortex were vulnerable to SL327 administration at P6 whereas those in subiculum were not. In this relation, it would be noteworthy that oligoapoptogenic action of alcohol depends on maturational stage of oligodendrocyte^18^.

Our results indicate that genetic abrogation of *Erk1* or *Erk2 per se* does not cause increased apoptosis at P6. Additionally, inhibition of both ERK1 and ERK2 may be required to induce increased apoptosis at P6. Although the mechanism is largely unknown, a possible explanation for these results is that ERK1 and ERK2 redundantly share their roles in apoptosis and compensate for each other, thus total phosphorylation levels of ERKs may be critical for the induction of apoptosis during the critical period. Consistently, the phosphorylation level of ERK2 is elevated in *Erk1* KO mice in a compensatory way, and vice versa as indicated in Fig. 12. There are many studies reporting that ERK1 and ERK2 play distinct roles in neuronal functioning. For instance, genetic abrogation of ERK2 has profound deleterious effects on neural functioning, whereas *Erk1* KO mice do not exhibit significant impairment in neural functioning^10^,^19^,^20^,^21^. In this respect, regulating mechanism of ERK in relation to the formation of neural circuits during the critical period may be different from that in the adult stage.

In *Erk2* CKO mice, the apoptosis level was significantly decreased when compared with control mice in the presence of SL327. Since ERK phosphorylation was almost completely blocked in the presence of SL327 both in control and *Erk2* CKO mice, there was no difference in the phosphorylation state between them. Thus, we speculated that developmental changes might occur in *Erk2* CKO mice before P6, which would alter the sensitivity to SL327-induced apoptosis. Consistent with this hypothesis, previous reports have indicated significant developmental changes by genetic abrogation of *Erk2*^22^,^23^,^24^, but not in *Erk1* KO mice^25^.

In humans, altered ERK activation causes a variety of developmental disorders which have ASD-like symptoms, although the pathogenic mechanisms are not precisely understood. For instance, mutations in different genes in the RAS/ERK pathway cause developmental disorders known as neuro-cardio-facial cutaneous (NCFC) syndrome, which is frequently associated with varying degrees of ASD-like symptoms^26^. Furthermore, altered expression of ERKs seems to be related to ASD-like symptoms. The gene encoding ERK2 is located on the distal region of chromosome 22q11.2. Deletion of chromosome 22q11.2 is the most frequent known interstitial deletion. It was reported that ASD is common in patients with 22q11.2 deletion syndrome^27^. The gene encoding ERK1 is located on 16p11.2. Deletion of chromosome 16p11.2 is also frequently associated with ASD^28^.

We previously reported that genetic abrogation of *Erk2* gene in the CNS results in ASD-like behavior in mice^10^. In the current study, we demonstrate that only transient inactivation of ERKs phosphorylation during the critical period cause ASD-like behaviors later in adulthood. Accumulating evidence suggests that ASD may result from disruption of proper formation of neural circuits during the critical period (see review^29^). Together, with the results in the current study, it is intriguing to speculate that altered ERKs activation might play important roles in the pathology of a broad range of neurodevelopmental disorders including ASD. Our results provide novel insight into the mechanistic basis for neurodevelopment disorders, and suggest that various neurodevelopment disorders might be generally linked to defects in ERKs signaling during the critical period. It might be important to keep ERK activation during the critical period for preventing developmental/mental retardation disorders. In this respect, drugs that can have an inhibitory effect on ERK activation, although long considered safe for pediatric use as with general anesthetics, might have potential to cause developmental/mental retardation disorders.

## Materials and Methods

### Ethics statement

The care and use of all mice in this study was carried out in accordance with institutional ethical guidelines for animal experiments and the safety guidelines for gene manipulation experiments of the National Defense Medical College (Tokorozawa, Saitama, Japan). All experimental protocols were approved by the Committee for Animal Research at the National Defense Medical College.

### Animals

*Erk2* CKO mice^10^ and *Erk1* KO mice^25^ were generated as described previously. Both *Erk1* and *Erk2* mutant mice were backcrossed with C57BL/6 for more than 10 generations. Adult mice were housed three or four per standard shoebox breeding cage (approx. size of 170 mm x 290 mm, 150 mm high) with autoclaved wood-chip bedding materials. Mice were maintained on a 12-h light-dark cycle (lights on from 07:00 to 19:00) with room temperature at 22 ± 1 °C with 46 ± 2% humidity. Mice had ad libitum access to water and food.

### MEK inhibitor administration

ERK is activated through the phosphorylation of tyrosine and threonine residues by immediate upstream kinase MEK. In the current study, ERK phosphorylation was inhibited by the blood-brain barrier-penetrating MEK inhibitor, SL327 (ENZO, Farmingdale, NY). SL327 was dissolved in dimethyl sulfoxide (DMSO; final concentration: 25 μg/μL). SL327 was administered intraperitoneally in a single dose at the concentration of 50 mg/kg to wild type, *Erk1* KO, and *Erk2* CKO mice at P6 or P14. This means that, if the body weight of the mouse was 5 g, then 10 μl of SL327 solution was injected. Control animals were given an injection of the same volume of DMSO.

### Western blot analysis

Western blot analysis of forebrain was performed as previously described^10^. Primary antibodies used were anti-cleaved poly-(adenosine diphosphateribose) polymerase (PARP) (#9544, rabbit polyclonal; Cell Signaling Technology, Beverly, MA), anti-ERK1/2 (#9102, rabbit polyclonal, Cell Signaling Technology), anti-phospho ERK1/2 (#9101, rabbit polyclonal, Cell Signaling), or anti-β-actin (A5441, mouse monoclonal, Sigma, St. Louis, MO). Secondary antibodies used were horseradish peroxidase (HRP)-linked anti-rabbit IgG (#7074, goat polyclonal, Cell Signaling Technology) and HRP-linked anti-mouse IgG (#7076, horse polyclonal, Cell Signaling Technology).

### Immunohistochemical studies

Immunohistochemical studies were performed as previously described^30^. Briefly, paraffin sections (5 μm thick) were de-paraffinized and immersed in unmasking solution (Vector H3300; Vector Laboratories, Burlingame, CA) for antigenic retrieval, and heated in an autoclave (121 °C) for 5 min. The sections were then incubated in a non-specific blocking reagent (Dako, Glostrup, Denmark) for 30 min to reduce background staining. Sections were then incubated with primary antibodies overnight in a humidified chamber at 4 °C. Primary antibodies used in this study were anti-active caspase-3 antiserum (#9661, rabbit polyclonal, Cell Signaling Technology), anti-NeuN (MAB377, mouse monoclonal, Millipore, Billerica, MA), anti-GFAP (G3893, mouse monoclonal, Sigma-Aldrich, St. Louis, MO), and anti-CNPase (MAB326R, mouse monoclonal, Millipore) antibodies.

In bright field dye staining, sections were incubated with peroxidase-conjugated secondary antibody (Dako EnVision+system; Dako). Immunoreactivity was revealed using 3,3-diaminobenzine-tetrachloride (DAB, Vector Laboratories) according to the manufacturer’s instruction. Finally, the sections were counter-stained with hematoxylin. In fluorescent staining, sections were incubated with Alexa-Fluor 488-conjugated goat anti-rabbit IgG (Molecular Probes, Eugene, OR) for primary antibodies derived from mouse. For primary antibodies derived from rabbit, Alexa-Fluor 546-conjugated goat anti-mouse IgG (Molecular Probes) was used. Sections were examined using a Nikon fluorescence microscopy system (Nikon, Tokyo, Japan) with electron-multiplying (EM) CCD digital camera (ImagEM, Hamamatsu Photonics, Hamamatsu, Japan). Counting of immunostaining was performed by an investigator blinded to the treatment conditions. Samples from at least five mice per experimental condition were examined.

### LTP measurement

Hippocampal slices were prepared from male C57BL/6 mice at 4 weeks of age, as previously described^31^. Briefly, after animals were decapitated under halothane anesthesia, the hippocampi were rapidly removed. Transverse slices (400 μm thick) were cut using a Vibratome 3000 (Vibratome, St. Louis, MO) in cold (2.5 °C) sucrose Ringer’s solution containing (in mM): 234 sucrose, 26 NaHCO_3_, 2.5 KCl, 0.5 CaCl_2_, 1.25 NaH_2_PO_4_, 10 MgSO_4_, and 11 glucose (pH 7.3–7.4), oxygenated with 95% O_2_/5% CO_2_. The slices were incubated in artificial cerebrospinal fluid (ACSF) containing (in mM): 26 NaHCO_3_, 124 NaCl, 3.0 KCl, 2.0 CaCl_2_, 1.2 KH_2_PO_4_, 1.3 MgHPO_4_·7H_2_O, and 10 glucose (pH 7.3–7.4) at room temperature for at least 90 min before use. A single slice was transferred to the recording chamber and perfused with ACSF at a rate of 2–3 mL/min (30 ± 2 °C). ACSF was continuously bubbled with 95% O_2_/5% CO_2_.

Field excitatory postsynaptic potentials (fEPSPs) were recorded from the stratum radiatum of the CA1 hippocampal area using a glass micropipette (1–2 MΩ) filled with ACSF. Electrical signals were amplified using an Axon Instruments MultiClamp 700B amplifier (Axon Instruments, Union City, CA), filtered at 10 kHz, digitized at 10 kHz, and acquired with Clampex software (version 9.2, Axon Instruments). A bipolar stainless steel stimulating electrode was placed in the stratum radiatum to stimulate the Schaffer collateral pathway. In all experiments, stimulus intensity was adjusted to produce a fEPSP that was 40–50% of the maximal amplitude. The duration of the stimulus was 0.10–0.15 ms. The strength of synaptic transmission was determined by measuring the onset slope (20–60% rising phase) of the fEPSP. The average fEPSP slope during the 10 min before LTP induction was taken as baseline, and all values were normalized to this baseline (normalized fEPSP slope). The average of the normalized fEPSP slope from 55 to 60 min after induction was taken as a measure of the maintenance of LTP. The stimulus frequency during the baseline was 0.033 Hz. LTP was induced by high frequency stimulation consisting of single 1-s trains of stimuli delivered at 100 Hz delivered. PPF was induced by delivering two pulses with a 20-, 50-, 70-, 100-, or 200-ms interpulse-interval.

### NMDA/AMPA ratio measurement

Hippocampal slices were prepared from male C57BL/6 mice at 4-5 weeks of age. After animals were decapitated under isoflurane anesthesia, the hippocampi were rapidly removed (< 30 sec) and placed immediately into ice-cold oxygenated (95% O_2_/5% CO_2_) cutting solution containing (in mM): 93 NMDG-Cl, 30 NaHCO_3_, 20 HEPES, 2.5 KCl, 10 MgSO_4_, 0.5 CaCl_2_, 1.2 NaH_2_PO_4_, 25 glucose, 5 sodium ascorbate, 3 sodium pyruvate and 2 thiourea (pH 7.4)^32^,^33^. Transverse hippocampus slices (350 μm-thick) were cut using a Linear Slice Pro 7 (Dosaka, Kyoto, Japan) in the cold cutting solution. The slices were incubated at 32-33 °C for 15–20 minutes in the cutting solution and then transferred into 22–24 °C normal artificial cerebrospinal fluid for patchclamp (ACSF-P) containing (in mM): 124 NaCl, 26 NaHCO_3_, 3 KCl, 2 MgCl_2_, 2 CaCl_2_, 1.25 NaH_2_PO_4_ and 11 glucose (pH 7.4).

Whole-cell recording pipettes (4–6 MΩ) were pulled from borosilicate glass and filled with a solution containing (mM): 120 Cs-gluconate, 20 CsCl, 10 NaCl, 10 HEPES, 0.2 EGTA, 4 MgATP, 0.3 Na_3_GTP and 5 phosphocreatine-diTris, pH 7.2 (adjusted with CsOH). Recordings were made with a CEZ-2400 amplifier (Nihon Kohden, Tokyo, Japan) and an A-D/D-A converter (25 kHz/channel, 16 bit; USB-6259BNC, National Instruments, Austin, USA). Hippocampal CA1 pyramidal cells were illuminated and visualized using an upright microscope (AxioSkop 2FS, Zeiss, Jena, Germany) equipped with a 40X water-immersion objective lens. The slices were constantly perfused with the ACSF-P with picrotoxin (50 μM) and glycine (50 μM) at ~2 ml/min at 31 ± 1 °C throughout the recordings.

The NMDA/AMPA current ratio provides a measure of NMDAR activity relative to AMPAR activity, regardless of the number of presynaptic afferents recruited and stimulating electrode positioning^34^. To evoke synaptic current in hippocampal CA1 pyramidal neurons, Schaffer collateral was stimulated by using a twisted wire electrode. Pyramidal neurons were voltage-clamped at -90 and + 40 mV to record AMPAR-mediated and NMDAR-mediated currents respectively. AMPAR-mediated index was measured at the peak of the current trace. NMDAR-mediated index was recorded 50 ms after the stimulation, when the short transient AMPA current had mostly disappeared^35^,^36^,^37^. Then, the NMDA/AMPA ratio was calculated by dividing the NMDA index by the AMPA index.

### Behavioral studies

Behavioral studies were performed as described previously^10^,^30^ at 9-11 weeks of age. Mice used in behavioral studies were age-matched male littermates except for studies of maternal behaviors. In each experiment, observation was made by the same observer who was blinded to the groups.

### Open-field test

Emotional responses to a novel environment were measured by the open-field test as previously described^16^. Activity was measured as the total distance traveled (meters) in 10 min.

### EPM test

The EPM test was used to measure anxiety-related behavior in rodents, as previously described^16^. Mouse behavior was recorded during a 10-min test period, and the percentage of time spent in the open arms was used as an index of anxiety-like behavior.

### Y-maze test

The Y-maze test was performed to assess spatial working memory, as previously described^16^. Each mouse was placed in the center of the Y-maze, and the mouse was allowed to freely explore the maze for 8 min. The total number of arms entered, and in which sequence, were recorded. The percentage of alternations was calculated as the number of triads containing entries into all three arms, divided by the maximum possible number of alternations (total number of arm entries minus 2)×100.

### Sociability test in the open-field chamber

The preference for interaction with animate (caged adult mouse) *versus* inanimate (caged dummy mouse) targets was examined in the open-field chamber, as previously described^16^. Briefly, animate or inanimate targets were put into cylindrical cages allowing olfactory but minimal tactile interaction. Sniffing directed at the cage was recorded for 10 min.

### Olfactory test

The olfactory test was conducted as previously described^16^. Briefly, mice were habituated to the flavor of a novel food (blueberry cheese) for 3 days prior to testing. On the fourth day, following 24-h food deprivation, a piece of blueberry cheese was buried under 2 cm of bedding in a clean cage. The mice were placed in the cage, and the time required to find the food was measured. The same sets of mice for the sociability test were used in the olfactory test.

### Novelty test

The novelty test was performed as previously described^16^. Mice were housed individually and activity was measured as the total time spent interacting with an inanimate novel object (a small red tube) in 10 min. The same sets of mice for the sociability test were used in the novelty test.

### Grooming behavior analysis

Self-grooming behavior was measured essentially as previously described^14^. Briefly, mice were habituated for 10 min in an empty cage without bedding, and then, grooming of all body regions was observed for 10 min.

### Arterial blood gas analysis

Arterial blood sampling from the left cardiac ventricle was performed as previously described^38^. Briefly, after anesthesia, arterial blood was obtained from the left cardiac ventricle and samples were analyzed using a blood gas analyzer (ABL800; Radiometer, Copenhagen, Denmark).

### Statistical analysis

Statistical analysis was performed using GraphPad Prism 6.0 (GraphPad Software Inc, La Jolla, CA). Comparisons of the means of each group were performed using a student’s t-test, a one-way analysis of variance (ANOVA) followed by the Bonferroni post hoc test, or a two-way ANOVA followed by the Bonferroni post hoc test. In the Y-maze test, comparisons of group performance versus random performance were carried out using a two-tailed one-sample t-test. Comparisons of the means of each group in LTP were performed using a two-way repeated measures ANOVA. *P* values of less than 0.05 were considered statistically significant. Values are presented as mean ± SEM.

## Author Contributions

S.Y., H.O. and I.T. performed the experiments; Y.S. designed the research; S.Y. and H.O. performed the statistical analysis; Y.S., Y.T., G.P. and J.P. generated the knockout mice; S.Y., Y.S. and Y.K. interpreted the data; S.Y., Y.S., H.O., S.E. and T.K. wrote the paper.

## Additional Information

**How to cite this article**: Yufune, S. *et al.* Transient Blockade of ERK Phosphorylation in the Critical Period Causes Autistic Phenotypes as an Adult in Mice. *Sci. Rep.* **5**, 10252; doi: 10.1038/srep10252 (2015).

## Figures and Tables

**Table 1:** No significant differences in pH, PaO_2_, and PaCO_2_ were observed among groups (one way ANOVA, *P* values > 0.05 for each; n = 7 mice for 0 h and 6 h (vehicle) group, n = 10 mice for 6 h (SL327) group. PaCO_2_: partial pressure of arterial carbon dioxide, PaO_2_: partial pressure of arterial oxygen.

	**0 h**	**6 h (vehicle)**	**6 h (SL327)**
pH	7.45 ± 0.03	7.26 ± 0.04	7.32 ± 0.06
PaO_2_ (mmHg)	133.9 ± 2.7	130.7 ± 2.4	134.2 ± 1.9
PaCO_2_ (mmHg)	34.7 ± 4.9	41.7 ± 5.1	37.5 ± 5.0

## References

[b1] Knudsen E. I. Sensitive periods in the development of the brain and behavior. J. Cogn. Neurosci. 16, 1412–1425 (2004). 15509387 10.1162/0898929042304796

[b2] Wiesel T. N. & Hubel D. H. Single-Cell Responses in Striate Cortex of Kittens Deprived of Vision in One Eye. J. Neurophysiol. 26, 1003–1017 (1963). 14084161 10.1152/jn.1963.26.6.1003

[b3] Nelson C. A. 3rd, *et al.* Cognitive recovery in socially deprived young children: the Bucharest Early Intervention Project. Science 318, 1937–1940 (2007). 18096809 10.1126/science.1143921

[b4] Ikonomidou C., Mosinger J. L., Salles K. S., Labruyere J. & Olney J. W. Sensitivity of the developing rat brain to hypobaric/ischemic damage parallels sensitivity to N-methyl-aspartate neurotoxicity. J. Neurosci. 9, 2809–2818 (1989). 2671294 10.1523/JNEUROSCI.09-08-02809.1989 PMC6569702

[b5] Ikonomidou C. *et al.* Blockade of NMDA receptors and apoptotic neurodegeneration in the developing brain. Science 283, 70–74 (1999). 9872743 10.1126/science.283.5398.70

[b6] Jevtovic-Todorovic V., *et al.* Early exposure to common anesthetic agents causes widespread neurodegeneration in the developing rat brain and persistent learning deficits. J. Neurosci. 23, 876–882 (2003). 12574416 10.1523/JNEUROSCI.23-03-00876.2003 PMC6741934

[b7] Satomoto M. *et al.* Neonatal exposure to sevoflurane induces abnormal social behaviors and deficits in fear conditioning in mice. Anesthesiology 110, 628–637 (2009). 19212262 10.1097/ALN.0b013e3181974fa2

[b8] Straiko M. M. *et al.* Lithium protects against anesthesia-induced developmental neuroapoptosis. Anesthesiology 110, 862–868 (2009). 19293695 10.1097/ALN.0b013e31819b5eab PMC2716053

[b9] Thomas G. M. & Huganir R. L. MAPK cascade signalling and synaptic plasticity. Nat. Rev. Neurosci. 5, 173–183 (2004). 14976517 10.1038/nrn1346

[b10] Satoh Y. *et al.* ERK2 contributes to the control of social behaviors in mice. J. Neurosci. 31, 11953–11967 (2011). 21849556 10.1523/JNEUROSCI.2349-11.2011 PMC6623182

[b11] Yon J. H., Daniel-Johnson J., Carter L. B. & Jevtovic-Todorovic V. Anesthesia induces neuronal cell death in the developing rat brain via the intrinsic and extrinsic apoptotic pathways. Neuroscience 135, 815–827 (2005). 16154281 10.1016/j.neuroscience.2005.03.064

[b12] Atkins C. M., Selcher J. C., Petraitis J. J., Trzaskos J. M. & Sweatt J. D. The MAPK cascade is required for mammalian associative learning. Nat. Neurosci. 1, 602–609 (1998). 10196568 10.1038/2836

[b13] Selcher J. C., Atkins C. M., Trzaskos J. M., Paylor R. & Sweatt J. D. A necessity for MAP kinase activation in mammalian spatial learning. Learn Mem. 6, 478–490 (1999). 10541468 10.1101/lm.6.5.478 PMC311312

[b14] Etherton M. R., Blaiss C. A., Powell C. M. & Sudhof T. C. Mouse neurexin-1alpha deletion causes correlated electrophysiological and behavioral changes consistent with cognitive impairments. Proc. Natl. Acad. Sci. USA 106, 17998–18003 (2009). 19822762 10.1073/pnas.0910297106 PMC2764944

[b15] Crawley J. N. Mouse behavioral assays relevant to the symptoms of autism. Brain pathology 17, 448–459 (2007). 17919130 10.1111/j.1750-3639.2007.00096.x PMC8095652

[b16] Satoh Y. *et al.* Extracellular signal-regulated kinase 2 (ERK2) knockdown mice show deficits in long-term memory; ERK2 has a specific function in learning and memory. J. Neurosci. 27, 10765–10776 (2007). 17913910 10.1523/JNEUROSCI.0117-07.2007 PMC6672813

[b17] Brambrink A. M. *et al.* Isoflurane-induced apoptosis of oligodendrocytes in the neonatal primate brain. Annals of neurology 72, 525–535 (2012). 23109147 10.1002/ana.23652 PMC3490441

[b18] Creeley C. E., Dikranian K. T., Johnson S. A., Farber N. B. & Olney J. W. Alcohol-induced apoptosis of oligodendrocytes in the fetal macaque brain. Acta neuropathologica communications 1, 23 (2013). 24252271 10.1186/2051-5960-1-23 PMC3893424

[b19] Otsubo Y. *et al.* Mechanical allodynia but not thermal hyperalgesia is impaired in mice deficient for ERK2 in the central nervous system. Pain 153, 2241–2252 (2012). 22902213 10.1016/j.pain.2012.07.020

[b20] Alter B. J., Zhao C., Karim F., Landreth G. E. & Gereau R.W.t. Genetic targeting of ERK1 suggests a predominant role for ERK2 in murine pain models. J. Neurosci. 30, 11537–11547 (2010). 20739576 10.1523/JNEUROSCI.6103-09.2010 PMC2932641

[b21] Mazzucchelli C. *et al.* Knockout of ERK1 MAP kinase enhances synaptic plasticity in the striatum and facilitates striatal-mediated learning and memory. Neuron 34, 807–820 (2002). 12062026 10.1016/s0896-6273(02)00716-x

[b22] Satoh Y. *et al.* Deletion of ERK1 and ERK2 in the CNS causes cortical abnormalities and neonatal lethality: Erk1 deficiency enhances the impairment of neurogenesis in Erk2-deficient mice. J. Neurosci. 31, 1149–1155 (2011). 21248139 10.1523/JNEUROSCI.2243-10.2011 PMC6632941

[b23] Samuels I. S. *et al.* Deletion of ERK2 mitogen-activated protein kinase identifies its key roles in cortical neurogenesis and cognitive function. J. Neurosci. 28, 6983–6995 (2008). 18596172 10.1523/JNEUROSCI.0679-08.2008 PMC4364995

[b24] Pucilowska J., Puzerey P. A., Karlo J. C., Galan R. F. & Landreth G. E. Disrupted ERK signaling during cortical development leads to abnormal progenitor proliferation, neuronal and network excitability and behavior, modeling human neuro-cardio-facial-cutaneous and related syndromes. J. Neurosci. 32, 8663–8677 (2012). 22723706 10.1523/JNEUROSCI.1107-12.2012 PMC6620980

[b25] Pages G. *et al.* Defective thymocyte maturation in p44 MAP kinase (Erk 1) knockout mice. Science 286, 1374–1377 (1999). 10558995 10.1126/science.286.5443.1374

[b26] Bentires-Alj M., Kontaridis M. I. & Neel B. G. Stops along the RAS pathway in human genetic disease. Nature medicine 12, 283–285 (2006). 10.1038/nm0306-283 16520774

[b27] Vorstman J.A. *et al.* Identification of novel autism candidate regions through analysis of reported cytogenetic abnormalities associated with autism. Molecular psychiatry 11, 118–28 (2006). 16205736 10.1038/sj.mp.4001781

[b28] Kolli S., Zito C.I., Mossink M.H., Wiemer E.A. & Bennett A.M. The major vault protein is a novel substrate for the tyrosine phosphatase SHP-2 and scaffold protein in epidermal growth factor signaling. J. Biol. Chem. 279, 29374–29385 (2004). 15133037 10.1074/jbc.M313955200

[b29] LeBlanc J. J. & Fagiolini M. Autism: a “critical period” disorder? Neural. Plast. 2011, 921680 (2011). 21826280 10.1155/2011/921680 PMC3150222

[b30] Yonamine R., Satoh Y., Kodama M., Araki Y. & Kazama T. Coadministration of hydrogen gas as part of the carrier gas mixture suppresses neuronal apoptosis and subsequent behavioral deficits caused by neonatal exposure to sevoflurane in mice. Anesthesiology 118, 105–113 (2013). 23221861 10.1097/ALN.0b013e318275146d

[b31] Takamatsu I., Sekiguchi M., Wada K., Sato T. & Ozaki M. Propofol-mediated impairment of CA1 long-term potentiation in mouse hippocampal slices. Neurosci. Lett. 389, 129–132 (2005). 16112456 10.1016/j.neulet.2005.07.043

[b32] Tanaka Y., Tanaka Y., Furuta T., Yanagawa Y. & Kaneko T. The effects of cutting solutions on the viability of GABAergic interneurons in cerebral cortical slices of adult mice. J. Neurosci. Methods 171, 118–125 (2008). 18430473 10.1016/j.jneumeth.2008.02.021

[b33] Zhao S. *et al.* Cell type-specific channelrhodopsin-2 transgenic mice for optogenetic dissection of neural circuitry function. Nature methods 8, 745–752 (2011). 21985008 10.1038/nmeth.1668 PMC3191888

[b34] Bagetta V. *et al.* Rebalance of striatal NMDA/AMPA receptor ratio underlies the reduced emergence of dyskinesia during D2-like dopamine agonist treatment in experimental Parkinson’s disease. J. Neurosci. 32, 17921–17931 (2012). 23223310 10.1523/JNEUROSCI.2664-12.2012 PMC6621675

[b35] Myme C. I. Sugino K., Turrigiano G. G. & Nelson S. B. The NMDA-to-AMPA ratio at synapses onto layer 2/3 pyramidal neurons is conserved across prefrontal and visual cortices. J. Neurophysiol. 90, 771–779 (2003). 12672778 10.1152/jn.00070.2003

[b36] Tozzi A. *et al.* Region- and age-dependent reductions of hippocampal long-term potentiation and NMDA to AMPA ratio in a genetic model of Alzheimer’s disease. Neurobiology of aging 36, 123–133 (2015). 25104560 10.1016/j.neurobiolaging.2014.07.002

[b37] Pilpel Y. *et al.* Synaptic ionotropic glutamate receptors and plasticity are developmentally altered in the CA1 field of Fmr1 knockout mice. The Journal of physiology 587, 787–804 (2009). 19103683 10.1113/jphysiol.2008.160929 PMC2669971

[b38] Kodama M. *et al.* Neonatal desflurane exposure induces more robust neuroapoptosis than do isoflurane and sevoflurane and impairs working memory. Anesthesiology 115, 979–991 (2011). 21956042 10.1097/ALN.0b013e318234228b

